# MicroRNA miR-324-3p Induces Promoter-Mediated Expression of RelA Gene

**DOI:** 10.1371/journal.pone.0079467

**Published:** 2013-11-12

**Authors:** Ashutosh Dharap, Courtney Pokrzywa, Shruthi Murali, Gopal Pandi, Raghu Vemuganti

**Affiliations:** 1 Department of Neurological Surgery, University of Wisconsin, Madison, Wisconsin, United States of America; 2 Neuroscience Training Program, University of Wisconsin, Madison, Wisconsin, United States of America; University of Massachusetts Medical, United States of America

## Abstract

MicroRNAs (miRNAs) are known to repress translation by binding to the 3’UTRs of mRNAs. Using bioinformatics, we recently reported that several miRNAs also have target sites in DNA particularly in the promoters of the protein-coding genes. To understand the functional significance of this phenomenon, we tested the effects of miR-324-3p binding to RelA promoter. In PC12 cells, co-transfection with premiR-324-3p induced a RelA promoter plasmid in a dose-dependent manner and this effect was lost when the miR-324-3p binding site in the promoter was mutated. PremiR-324-3p transfection also significantly induced the endogenous RelA mRNA and protein expression in PC12 cells. Furthermore, transfection with premiR-324-3p increased the levels of cleaved caspase-3 which is a marker of apoptosis. Importantly, the miR-324-3p effects were Ago2 mediated as Ago2 knockdown prevented RelA expression and cleavage of caspase-3. Thus, our studies show that miRNA-mediated transcriptional activation can be seen in PC12 cells which are neural in origin.

## Introduction

MicroRNAs (miRNAs) are a class of small, ∼22 nucleotides long, non-coding RNAs that are known to be well conserved during evolution [1], [2]. Although miRNAs are conventionally thought to regulate protein translation by targeting the 3’UTRs of mRNAs, recent studies have reported that miRNAs and synthetic double-stranded RNAs can also bind to the consensus seed sequences in the promoter regions within DNA and such interactions modulate gene expression [3], [4], [5], [6], [7], [8].

Promoter-targeting small duplex RNAs (dsRNAs) were initially shown to activate the expression of various genes such as progesterone receptor, E-cadherin, p21 and VEGF [6], [9]. A VEGF promoter-targeting shRNA that was stably introduced in mouse by lentiviral delivery resulted in increased vascularity and blood flow in an ischemic hind limb model via VEGF upregulation [10]. Subsequent studies provided further evidence of this phenomenon in various mammalian systems. Some prominent examples include the dsRNA-mediated activation of p53 in chimpanzee cells that resulted in PARP cleavage indicative of caspase-dependent apoptosis due to upregulation of p53, specific upregulation of the rat chemokine receptor CXCR4 by dsRNAs in adipose-derived stem cells, and Cyclin B1 induction by two specific activating RNAs that increased phosphorylation of histone H3 at Serine 10 correlating with mitotic chromosomal condensation (Cycline B1 promotes entry into mitosis) [3]. From a clinical standpoint, a recent study exploring a strategy to treat hypercholesterolemia showed that targeting of the low-density lipoprotein receptor (LDLR) by dsRNAs activated its expression and increased display on the surfaces of liver cells [11]. Another study showed that the specific overexpression of the transcription factor KLF4 by dsRNAs inhibited prostate cancer cell proliferation, survival and metastasis [12].

By genome-wide analysis of miRNA binding sites within the putative promoters of the whole rate genome, we observed many miRNA binding sites in protein-coding gene promoters [13]. One of the strongest interactions was observed to be between miR-324-3p and the RelA (commonly known as p65, a subunit of nuclear factor-κB; NF-κB). As NF-κB is a major contributor of inflammation following an insult to brain, understanding the factors responsible for its induction helps in controlling its activity [14].

We presently analyzed if miR-324-3p induces RelA mRNA and protein expression by interacting with RelA promoter in a sequence-specific manner. Argonaute (Ago) family of proteins, particularly Ago2 is an essential component of the RNA-induced silencing complex (RISC) that is mediates translational repression by miRNAs [15]. Hence, we analyzed if Ago2 also necessary for the miR-324-3p mediated transcriptional activation of RelA gene.

## Methods

### Cell Culture

PC12 adherent cells [American Type Culture Collection (ATCC)] were cultured as described earlier [16]. In brief, cells were maintained in high glucose DMEM medium (GIBCO USA) containing 4.5 g/L glucose, L-glutamine and 110 mg/mL sodium pyruvate supplemented with 10% inactivated horse serum. Cells were cultured at 37°C and 5% CO_2_. For measuring the effect of premiR-324-3p on the endogenous RelA RNA and protein expression, cells were plated in 10 cm^2^ dishes at a density of 2×10^6^ cells/dish. For promoter vector activation experiments, cells were plated in 24-well plates at a density of 1×10^5^ cells/well. The cell viability was analyzed by Trypan Blue exclusion assay.

### Plasmids

A 1 kb region of the RelA promoter was amplified from rat genomic DNA using the primers CCTTATTTTTCAGTAAGTACACTCATG (forward) and CCATTCGCCAGAGGC (reverse). The amplified product was cloned into a pGEM-T Easy vector (Promega USA) and the sequence was confirmed by automated DNA sequencing and then subcloned into promoter-less pGL3-Basic vector (Promega USA) at the KpnI site upstream to luciferase reporter gene by the infusion cloning method (Clontech USA). A RelA mutant promoter plasmid was generated by creating point mutations in the miR-324-3p binding site by site-directed mutagenesis kit (Invitrogen USA) as per manufacturer’s instructions. The sequence and orientation of the wild-type and mutant plasmids were confirmed by restriction digestion and automated DNA sequencing. The plasmids were propagated in E. coli and purified using the NucleoBond Xtra Midi Plus kit (Clontech USA). The Renilla luciferase vector was from Promega USA.

### Transfections

The premiR-324-3p and the control premiR were from Ambion USA. Ago2 siRNA was from Applied Biosystems USA and the RelA Sure Silence siRNA was from Dharmacon USA. All sequences were transfected into PC12 cells (1×10^6^ cells/well) using RNAiMAX (Invitrogen USA). The premiR and control miR were transfected at a concentration of 50 to 150 nM and the siRNAs were transfected at a concentration of 100 nM. To account for the non-sequence-specific effects, an Ambion control non-targeting siRNA with comparable GC content to that of the functional siRNA but lacking identity with known gene targets and had at least 4 mismatches with all known human, mouse and rat genes was used. Each transfection was conducted in triplicate and each experiment was repeated 4 times.

### Luciferase reporter assays

PC12 cells were transfected with 50 to 150 nM premiR-324-3p control premiR. After 24 hours, wild-type or mutant RelA plasmid was transfected at a concentration of 300 ng together with 100 ng Renilla luciferase plasmid (transfection control) using Lipofectamine 2000 (Invitrogen USA). Two days after transfection, cells were lysed and subjected to a dual luciferase assay (Promega USA). Each transfection was conducted in triplicate and each experiment was repeated 4 times.

### Real-time PCR

The reverse transcription was performed using the TaqMan® MiRNA Reverse Transcription Kit (Applied Biosystems USA) and the PCR reactions were performed using the SYBR Green method as described earlier [16], [17]. The sequences of the primer used to amplify RelA (NM_199267.2) were TCTGCTTCCAGGTGACAGTG (forward) and ATCTTGAGCTCGGCAGTGTT (reverse). 18S rRNA was used as the internal controls and its primer sequences are same as in a previous study from our lab [13].

### Western Blotting

Cells were homogenized in ice-cold 25 mM Tris-HCl buffer (pH 7.4) containing 2 mM EDTA and protease inhibitor cocktail (4-(2-aminoethyl) benzenesulfonyl fluoride, aprotinin, leupeptin, bestatin, pepstatin-A, and transepoxysuccinyl-L-leucylamido(4-guanidino)butane; Sigma Chemical Co. USA). Protein estimation was conducted by DC™ Protein Assay (Bio-Rad USA) as per manufacturer’s instructions. with Proteins were solubilized by adding Lamelli electrophoresis sample buffer (5% sodium dodecyl sulfate, 20% glycerol, 10% 2- mercaptoethanol, 125 mmol/L Tris-HCl, pH 6.8, and 0.004% bromophenol blue; Sigma Chemical Co. USA) and denatured by heating at 94°C for 3 min. Samples (25–35 µg protein equivalent) were electrophoresed on 4–20% polyacrylamide gradient gels (Bio-Rad USA Criterion precast gels), transferred to PVDF membranes and probed with monoclonal anti-NF-κB p65 (Cell Signaling Technology USA), monoclonal anti-Ago2/eIF2C2 (Abcam USA) and β-actin (Cell Signaling Technology USA) antibodies followed by HRP-conjugated anti-rabbit or anti-mouse IgG. The protein bands recognized by the antibodies were visualized by enhanced chemiluminescence according to the manufacturer’s instructions (Pierce USA).

## Results

### miR-324-3p targets RelA promoter to induce its expression

**Figure 1:**
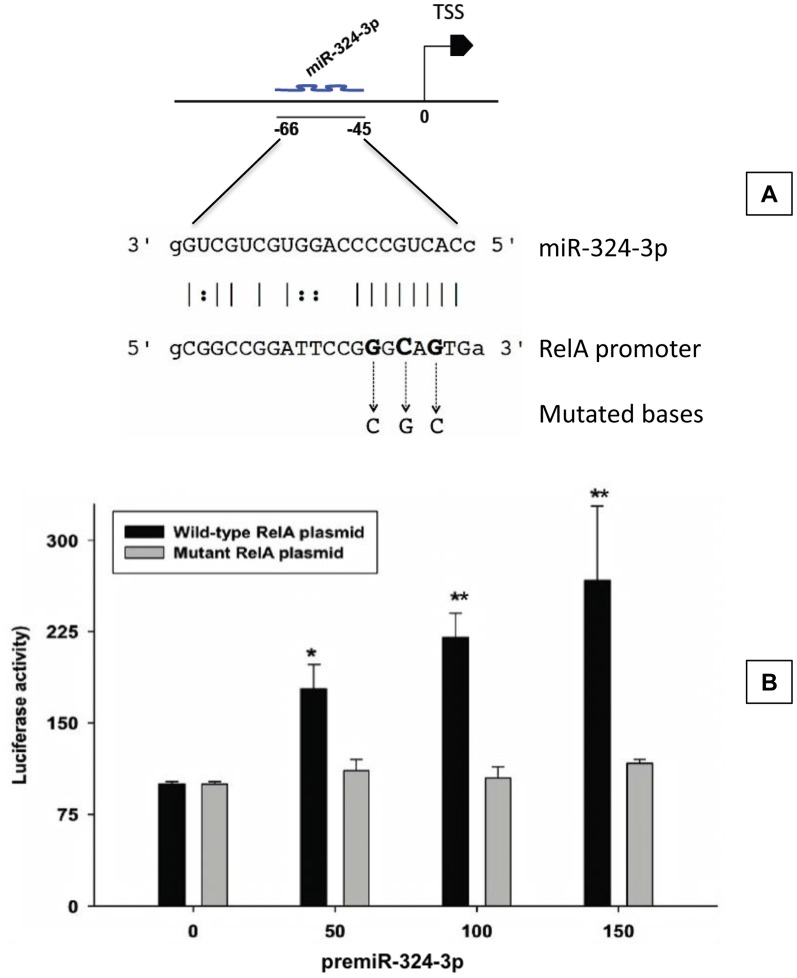
miR-324-3p induced RelA promoter. A miR-324-3p binding sits is present at nucleotides 45 to 66 upstream to RelA transcription start site (TSS) (A). Three bases (shown in bold) in the miR-324-3p binding site were mutated in the RelA promoter to create a mutant promoter vector (A). The RelA promoter vector showed significant dose-dependent induction when cotransfected with premiR-324-3p (B). The RelA mutant vector showed no induction by premiR-324-3p (B). Bars represent mean ± SD (n  =  4/group). Each assay was conducted in triplicate. *p<0.05 and **p<0.01 compared to the respective mutant group (Student’s t-test).

Using RegRNA algorithm, we previously showed that many miRNAs have binding sites in gene promoters in DNA [13]. Of particular interest, the interaction between miR-324-3p and RelA promoter showed a very high stringency (minimal free energy of –39.5 and a score of >155). The miR-324-3p target site was also observed to be located at the beginning of the RelA promoter (nucleotides 45 to 66 upstream to the transcription start site; TSS) (Fig. 1A). To confirm the miR-target functionality, we cloned a 1,000 kb region of the wild type RelA promoter from the rat genomic DNA upstream to an inducible firefly luciferase gene in promoterless pGL3 plasmid. We also constructed a mutant plasmid by disrupting the miR-324-3p site in the RelA promoter. When co-transfected, the premiR-324-3p induced RelA promoter expression in a dose-dependent manner compared to mutant vector (50 nM, 100 nM and 150 nM premiR-324 induced the luciferease activity by 178%, 220% and 267%, respectively; p<0.05) (Fig. 1B). The mutant RelA plasmid in which the miR-324-3p binding site was disrupted showed no induction when co-transfected with 50 to 150 nM premiR-324-3p (Fig. 1B). Renilla luciferase was used to normalize the RelA expression values.

### miR-324-3p induced endogenous RelA expression in an Ago2 dependent manner

**Figure 2:**
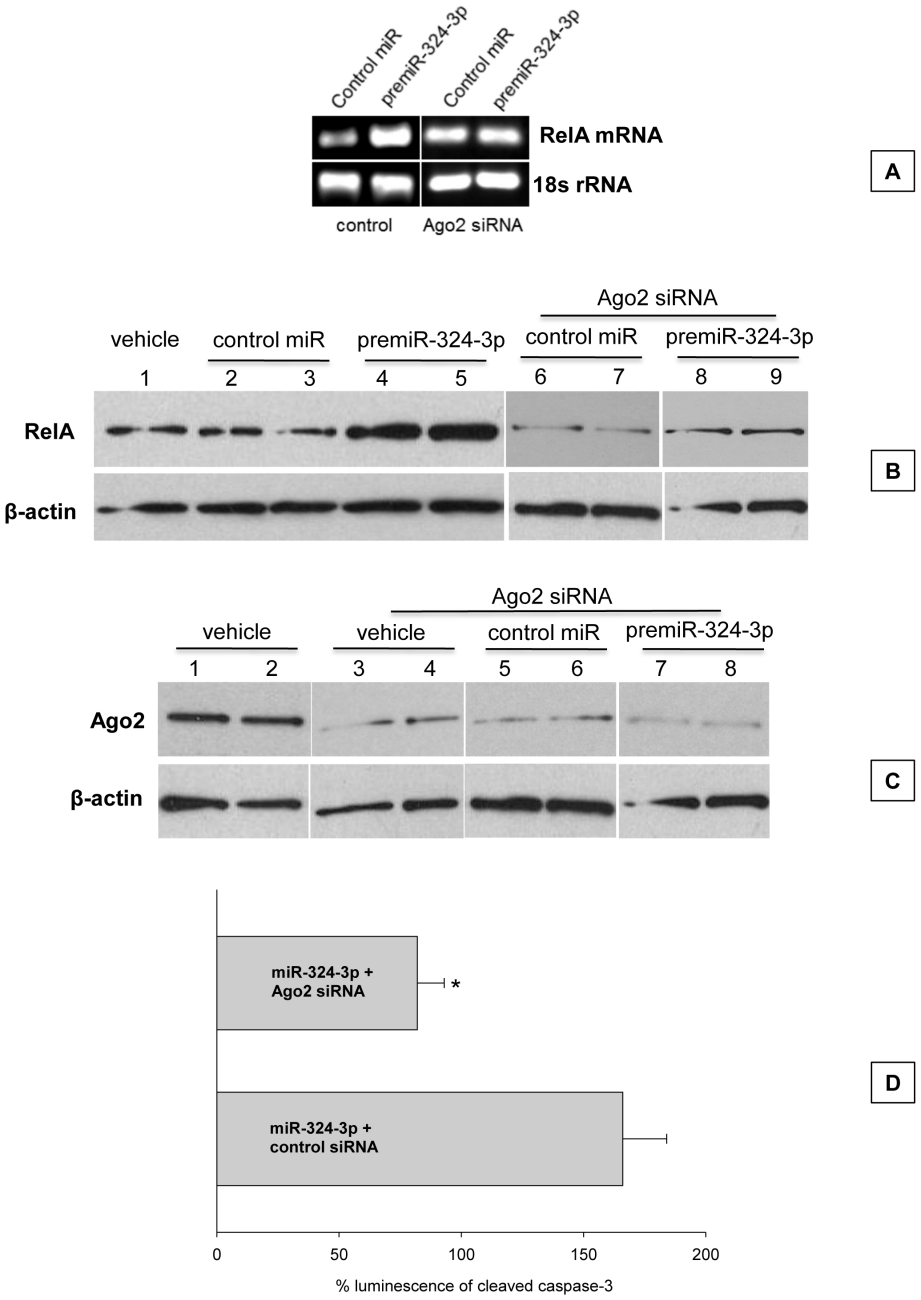
Induction of endogenous RelA by miR-324-3p. When PC12 cells were transfected with 150 nM of premiR-324-3p, there was a significant induction of RelA mRNA expression compared to control premiR treated group at 3 days after transfection (A). Ago2 siRNA treatment prevented the RelA induction by miR-324-3p. The house-keeping control 18S rRNA expression was not changed by premiR-324-3p transfection or Ago2 knockdown (A). PremiR-324-3p transfection also led to significant increase in the RelA protein levels compared to control miR treated or vehicle treated control groups (B). Treatment with Ago2 siRNA prevented the premiR-324-3p mediated increase in RelA protein levels (B). The levels of β-actin used as a loading control were not affected by premiR-324-3p or Ago2 siRNA (B). The efficiency of Ago2 siRNA to knockdown Ago2 protein levels in PC12 cells in the presence and absence of premiR-324-3p and control miR was confirmed by Western blotting. The Ago2 siRNA knocked-down Ago2 protein levels by >85% in all the 3 groups (treated with vehicle or control miR or premiR-324-3p) compared to vehicle treated group (C). PremiR-324-3p transfection induced significant activation of caspase-3 as measured by cleaved caspase-3 levels in PC12 cells which was prevented by Ago2 knockdown (D). Bars represent mean ± SD of n  =  4/group of triplicate determinations. * p<0.05 compared with the control siRNA group (Student’s t-test).

PremiR-324-3p (150 nM) transfection significantly increased the endogenous RelA mRNA levels compared to control premiR transfected cells in PC12 cells (Fig. 2A). We tested if Ago2 is an essential component for the miRNA-induced gene activation similar to its role in miRNA-induced translational repression. When Ago2 was knocked-down with a specific siRNA, premiR-324-3p was unable to induce RelA mRNA expression compared to control miR treated cells (Fig. 2A). The premiR-324-3p transfected cells also showed significantly higher RelA protein levels compared to control miR treated cells and Ago2 knockdown significantly curtailed RelA protein induction by premiR-324-3p (Fig. 2B). We confirmed that the siRNA used efficiently knocked-down Ago2 in the presence or absence of premiR-324-3p (Fig. 2C). RelA induction is a known promoter of apoptosis and transfection with premiR-324-3p elevated the levels of cleaved caspase-3 (Fig. 2D). Furthermore, Ago2 siRNA significantly prevented cleaved caspase-3 increase in miR-324-3p transfected cells (Fig. 2D).

## Discussion

Several recent reports have shown that miRNAs and other double-stranded RNAs can bind to the consensus seed sequences in the gene promoters within DNA and such interactions activate gene expression with significant effects on the transcriptome and proteome in many types of mammalian cells [3], [4], [5], [6], [7], [8]. However, this phenomenon has not yet been demonstrated in the neural cells. Using bioinformatics, we previously showed that many stroke-responsive miRNAs have binding sites in promoters of protein-coding genes [13]. Of those, RelA promoter showed a very high stringency bindings site for miR-324-3p. RelA is a major subunit of the pro-inflammatory transcription factor NF-κB protein complex that plays a significant role in neuronal death following acute insults to CNS like ischemic stroke.

All 5 subunits of NF-κB are known to be activated after cerebral ischemia, but they differ in their functional significance [18], [19], [20]. The c-Rel subunit has been shown to play a protective role, whereas RelA activation overrides that resulting in increased neuronal damage after ischemia [20]. When RelA expression was inhibited using conditional RelA mutants, there was a reduction in infarct volume following cerebral ischemia similar to that seen in mice lacking p50 suggesting that the p50/RelA heterodimers or RelA/RelA homodimers promote ischemic brain damage [18], [21]. Despite the importance of RelA in cerebral homeostasis, the mechanisms that control RelA have not been studied in detail. Based on the in silico analysis, we predicted that RelA expression is under the control of miR-324-3p. Interestingly miR-324-3p was one of the miRNAs induced as early as 6h after focal cerebral ischemia and sustained at high levels at least up to 3 days [13]. This indicates that miR-324-3p might be responsible in part for the induction of RelA after ischemia. Our present studies provide evidence for this. We confirmed that miR-324-3p induced RelA promoter plasmid dose-dependently in a miR-324-3p binding site specific manner. We further showed that miR-324-3p induces endogenous RelA mRNA and protein expression. We also showed that this is a miRNA-specific effect as knockdown of Ago2 which is critical for miRNA function prevented miR-324-3p induced increase in RelA mRNA and protein levels. As RelA induction promotes apoptosis, we further showed that miR-324-3p induces significant caspase-3 activation which was prevented by Ago2 knockdown.

Although the mechanisms of RNA-induced transcriptional regulation is largely unclear, recent studies suggested that non-coding (nc) RNAs activate transcription by removing repressive entities or promoting activating epigenetic marks. It was shown that promoter-overlapping ncRNA transcripts that usually repress transcription of the locus are targets of antigene RNAs (agRNAs), which either degrade the ncRNA transcripts to activate transcription or form an intact transcript-Ago-agRNA complex that acts as a scaffold to recruit other proteins and RNA Polymerase II at the target site to establish activating chromatin marks and induce transcription [9], [22], [23], [24]. We observed that miR-324-3p targets the RelA promoter in close proximity (within 100 bp) of the transcription start site (TSS) suggesting that the Ago2-miR-324-3p complex might recruit other proteins such as EZHs to open chromatin and activate transcription at the TSS. EZHs are chromatin modifiers that are associated with transcriptional activation. EZH1 was found to extensively overlap with the H3K4me3 activating promoter mark and RNAP II in thousands of genes during skeletal muscle differentiation [25]. Similarly, EZH1 was associated with increased gene transcription and RNAP II at the PSD-95 gene promoter in developing hippocampal neurons [26]. By interacting with the Ago2-miR-324-3p complex proteins such as EZHs might enable a transcriptional response. Other examples of small RNA based transcriptional activation also focus on epigenetic modifications such as activation of the VEGF promoter by small ncRNAs via an increase in H3K4me3 levels and a decrease in the repressive mark H3K9me2, thereby resulting in a more accessible chromatin architecture [6]. Hence, we speculate that a potential mode of action for the Ago2-miR-324-3p complex to induce the expression of RelA may be via recruitment of chromatin-modifying proteins to establish activating chromatin signatures and decreasing repressive marks in order to make the site amenable to transcriptional activation.

In conclusion our studies show that miRNA-mediated promoter-based transcriptional activation also occurs in cells of neural origin and further they play a functional role in modulating the activity of the down-stream targets. Thus, miRNA therapeutics can be used to control pathological consequences of injury like inflammation and cell death in CNS.
